# Trends in Sexual Harassment Prevalence and Recognition During Intern Year

**DOI:** 10.1001/jamahealthforum.2024.0139

**Published:** 2024-03-22

**Authors:** Elena Frank, Zhuo Zhao, Yu Fang, Jennifer L. Cleary, Elizabeth M. Viglianti, Srijan Sen, Constance Guille

**Affiliations:** 1 Michigan Neuroscience Institute, University of Michigan, Ann Arbor, Michigan; 2 Department of Psychology, University of Michigan, Ann Arbor, Michigan; 3 Division of Pulmonary and Critical Care Medicine, University of Michigan, Ann Arbor, Michigan; 4 Department of Psychiatry, University of Michigan Medical School, Ann Arbor, Michigan; 5 Department of Psychiatry and Behavioral Sciences, Medical University of South Carolina, Charleston, South Carolina

## Abstract

This cohort study uses Internal Health Study and Sexual Experiences Questionnaire data to assess changes in sexual harassment prevalence and recognition among training physicians.

## Introduction

#MeToo went viral in October 2017, increasing cultural consciousness of sexual harassment. Training physicians’ experience of sexual harassment has been associated with adverse mental health, poorer patient care, and career attrition.^1,2,3,4,5^ Yet, evaluation of the prevalence and trends in sexual harassment during medical training using established measures is limited, and rates of recognition are unknown. This study assesses changes in sexual harassment prevalence and recognition among interns from 2017 to 2023.

## Methods

This cohort study uses survey data from physicians enrolled in the longitudinal Intern Health Study in 2016, 2017, and 2022. The University of Michigan institutional review board approved the study. Participants provided electronic informed consent and were compensated $25 to $130. The study followed the AAPOR reporting guideline.

Participants completed a follow-up survey during the last month of internship (June 2017, 2018, and 2023) that assessed sexual harassment using a single-item self-report question and the 19-item Sexual Experiences Questionnaire-Shortened (SEQ-S), a validated, behavior-based instrument that assesses 3 types of sexual harassment: gender harassment, unwanted sexual attention, and sexual coercion^2,6^ (eMethods 2 in Supplement 1). Frequency of each item is indicated on a 5-point Likert scale (0 = never, 4 = very often).

We generated poststratification weights such that the distribution of cohort year, sex, specialty, and self-reported race and ethnicity from the preinternship survey matched the overall characteristics of US interns, reducing possible nonrepresentative sampling, and overlap weights to reduce participation attrition biases (eMethods 1 in Supplement 1). Participants who endorsed at least 1 item of the SEQ-S were considered to have experienced sexual harassment.^2^ To assess recognition, we compared the proportion of participants indicating sexual harassment on the SEQ-S with those who responded yes on the self-report question. Logistic regression models were performed to assess changes over time in sexual harassment prevalence and recognition, controlling for age, sex, race and ethnicity, and specialty. Analyses were conducted using SAS, version 9.4 (SAS Institute Inc). Two-sided *P* < .05 was considered significant.

## Results

Overall, 4178 interns completed the sexual harassment questions (median [IQR] age, 27 [26-28] years; 2159 women [51.7%]; 2019 men [48.3%]) (Table 1). After sample weighting, from 2017 to 2023, sexual harassment incidence decreased (from 62.8% to 54.6%; odds ratio [OR], 0.92; 95% CI, 0.90-0.94). Gender harassment incidence decreased (from 61.0% to 51.7%; OR, 0.91; 95% CI, 0.89-0.93), while sexual coercion incidence increased for women (from 2.3% to 5.5%; OR, 1.17; 95% CI, 1.08-1.28) and nonsurgical interns (from 1.6% to 4.0%; OR, 1.18; 95% CI, 1.09-1.27). Recognition of sexual harassment increased (from 8.6% to 18.4%; OR, 1.12; 95% CI, 1.07-1.17). This change was greater among women vs men and among surgical vs nonsurgical interns. Gender harassment recognition increased overall (from 8.9% to 18.9%; OR, 1.12; 95% CI, 1.07-1.17). Unwanted sexual attention recognition increased among women (from 29.7% to 41.8%; OR, 1.09; 95% CI, 1.03-1.16) and surgical interns (from 18.8% to 52.5%; OR, 1.27; 95% CI, 1.12-1.44) (Table 2).

**Table 1.** Sample Demographic Characteristics

Characteristic	No. of participants (%)	
	Unweighted (n = 4178*)*	Weighted (n = 4460)
Age, median (IQR), y	27 (26-28)	27 (26-28)
Sex		
Female	2159 (51.7)	2162 (48.5)
Male	2019 (48.3)	2298 (51.5)
Race and ethnicity		
American Indian or Alaska Native	2 (0.1)	2 (0.04)
Arab or Middle Eastern	60 (1.4)	82 (1.8)
Asian	820 (19.6)	1074 (24.1)
Black or African American	151 (3.6)	214 (4.8)
Latino or Hispanic	138 (3.3)	169 (3.8)
Native Hawaiian or Pacific Islander	4 (0.1)	3 (0.1)
White	2587 (61.9)	2356 (52.8)
Multiracial	394 (9.4)	536 (12.0)
Other^a^	22 (0.5)	24 (0.5)
Specialty		
Internal medicine	978 (23.4)	1155 (25.9)
Pediatrics	553 (13.2)	529 (11.9)
Emergency medicine	371 (8.9)	396 (8.9)
General surgery	373 (8.9)	448 (10.1)
Family medicine	330 (7.9)	350 (7.9)
Psychiatry	294 (7.0)	287 (6.4)
Obstetrics and gynecology	247 (5.9)	226 (5.1)
Anesthesiology	215 (5.2)	244 (5.5)
Neurology	110 (2.6)	132 (3.0)
Internal medicine–pediatrics	100 (2.4)	108 (2.4)
Otolaryngology	55 (1.3)	57 (1.3)
Transitional	154 (3.7)	145 (3.3)
Other	398 (9.5)	383 (8.6)

^a^ Interns self-reported as “other.”

**Table 2.** Trends in Prevalence and Recognition of Sexual Harassment From 2017 to 2023

Category	No. (%)^a^			Odds ratio (95% CI)	*P* value
	2017^b^	2018	2023	Change across 6 y	
**Incidence**					
Overall					
All interns	795 (62.8)	880 (64.4)	997 (54.6)	0.92 (0.90-0.94)	<.001
Women	450 (76.6)	492 (76.8)	671 (72.0)	0.96 (0.92-0.99)	.01
Men	345 (50.8)	388 (53.4)	326 (36.4)	0.90 (0.87-0.92)	<.001
Surgical^c^	164 (67.1)	171 (66.4)	197 (57.5)	0.91 (0.86-0.96)	.001
Nonsurgical	631 (61.7)	709 (63.9)	800 (53.9)	0.92 (0.90-0.95)	<.001
Gender harassment^d^					
All interns	773 (61.0)	853 (62.4)	944 (51.7)	0.91 (0.89-0.93)	<.001
Women	441 (75.0)	483 (75.4)	647 (69.4)	0.95 (0.92-0.98)	.002
Men	332 (48.9)	370 (51.0)	297 (33.2)	0.89 (0.86-0.91)	<.001
Surgical^c^	159 (65.1)	161 (62.5)	182 (53.4)	0.90 (0.85-0.95)	<.001
Nonsurgical	614 (60.0)	692 (62.4)	762 (51.3)	0.92 (0.89-0.94)	<.001
Unwanted sexual attention^e^					
All interns	242 (19.1)	293 (21.5)	387 (21.4)	1.00 (0.98-1.03)	.78
Women	167 (28.4)	200 (31.2)	263 (28.7)	0.99 (0.96-1.03)	.57
Men	75 (11.0)	93 (12.9)	124 (13.8)	1.03 (0.98-1.08)	.23
Surgical^c^	47 (19.3)	48 (18.5)	79 (23.5)	1.04 (0.97-1.10)	.27
Nonsurgical	195 (19.0)	245 (22.1)	308 (20.9)	1.00 (0.97-1.03)	.84
Sexual coercion^f^					
All interns	24 (1.9)	27 (1.9)	67 (3.7)	1.12 (1.05-1.20)	<.001
Women	14 (2.3)	14 (2.1)	50 (5.5)	1.17 (1.08-1.28)	<.001
Men	11 (1.6)	13 (1.8)	17 (1.9)	1.05 (0.93-1.17)	.45
Surgical^b^	9 (3.5)	8 (2.9)	8 (2.5)	0.93 (0.80-1.09)	.35
Nonsurgical	16 (1.6)	19 (1.7)	59 (4.0)	1.18 (1.09-1.27)	<.001
**Recognition**					
Overall					
All interns	69 (8.6)	103 (11.7)	183 (18.4)	1.12 (1.07-1.17)	<.001
Women	59 (13.1)	84 (17.0)	161 (23.9)	1.12 (1.07-1.17)	<.001
Men	10 (2.8)	19 (5.0)	22 (6.9)	1.17 (1.05-1.30)	.006
Surgical^b^	11 (6.6)	16 (9.3)	53 (26.7)	1.30 (1.18-1.43)	<.001
Nonsurgical	58 (9.2)	87 (12.3)	130 (16.3)	1.08 (1.03-1.14)	.001
Gender harassment^d^					
All interns	69 (8.9)	100 (11.8)	179 (18.9)	1.12 (1.07-1.17)	<.001
Women	59 (13.4)	83 (17.1)	156 (24.1)	1.12 (1.06-1.17)	<.001
Men	10 (2.9)	18 (4.8)	22 (7.6)	1.19 (1.06-1.32)	.003
Surgical^b^	11 (6.8)	15 (9.4)	53 (28.8)	1.32 (1.19-1.45)	<.001
Nonsurgical	58 (9.4)	85 (12.3)	126 (16.5)	1.08 (1.03-1.14)	.002
Unwanted sexual attention^e^					
All interns	58 (23.9)	84 (28.8)	132 (34.2)	1.08 (1.03-1.14)	.004
Women	50 (29.7)	66 (33.3)	110 (41.8)	1.09 (1.03-1.16)	.005
Men	8 (10.9)	18 (19.2)	22 (18.1)	1.11 (0.98-1.26)	.11
Surgical^b^	9 (18.8)	12 (25.6)	42 (52.5)	1.27 (1.12-1.44)	<.001
Nonsurgical	49 (25.1)	72 (29.4)	90 (29.5)	1.04 (0.98-1.11)	.18
Sexual coercion^f^					
All interns	10 (42.0)	6 (23.6)	26 (38.1)	1.01 (0.86-1.20)	.88
Women	8 (54.7)	4 (29.2)	26 (50.8)	1.12 (0.92-1.37)	.25
Men	3 (25.8)	2 (17.7)	0	0.58 (0.23-1.44)	.24
Surgical^b^	4 (43.1)	1 (17.8)	5 (61.6)	1.07 (0.63-1.83)	.81
Nonsurgical	6 (41.4)	5 (25.9)	21 (34.8)	0.97 (0.80-1.17)	.72

^a^ Interns could indicate more than 1 type of sexual harassment; therefore, percentages do not add to 100%.

^b^ The assessment was completed in June each year, during the last month of internship.

^c^ Surgical specialties were assigned based on the American College of Surgeons classification. Specifically, for this study, physicians in the following specialties were classified as surgical: general surgery, gynecology and obstetrics, neurologic surgery, orthopedic surgery, otolaryngology, plastic surgery, urology, and other surgical. Physicians from the following specialties were classified as nonsurgical: internal medicine, pediatrics, psychiatry, neurology, emergency medicine, internal medicine-pediatrics, family medicine, family practice, anesthesiology, dermatology, medical genetics, nuclear medicine, pathology, physical medicine and rehabilitation, preventive medicine, radiation oncology, radiology-diagnostic, sleep medicine, and other nonsurgical.

^d^ Verbal and nonverbal behaviors that convey hostility, objectification, exclusion, or second-class status about members of a gender.

^e^ Verbal or physical unwelcome sexual advances, which can include assault.

^f^ When favorable professional or educational treatment is conditioned on sexual activity.

## Discussion

The prevalence of training physicians’ experience of sexual harassment has decreased over time, while recognition has increased. The significant rise in gender harassment recognition, including a 4-fold increase among surgical interns, suggests that awareness has improved within medicine. The finding that sexual coercion prevalence has doubled is concerning.^2^ In 2023, 3 of 4 female interns experienced sexual harassment, and 1 of 4 identified their experiences as such. This gap between experience and recognition may reflect the extent to which sexual and gender-based discriminatory behavior remains ingrained in the culture of medicine. Thus, attention must shift beyond organizational policy compliance to address climate issues unique to institutions and specialties.^1^

Limitations include underreporting and potentially influential events beyond #MeToo during the study period. Future studies should explore the role of specialty-, institution-, and program-level factors, including harassment source. As sexual harassment has serious implications for physician well-being, performance, and retention, system-wide efforts must foster a more healthy and equitable culture within medicine.

## Notes

Supplement 1. **eMethods 1.** Sample Weighting Strategy
eReferences
**eMethods 2.** Survey Questions

Supplement 2.
Data Sharing Statement
