# Supplementary material for: Trends in Sexual Harassment Prevalence and Recognition During Intern Year
Source: JAMA Health Forum. 2024 Mar 22;5(3):e240139. doi: 10.1001/jamahealthforum.2024.0139 (PMC10960195; doi:10.1001/jamahealthforum.2024.0139)
Supplement: Supplement 1. — eMethods 1. Sample Weighting Strategy eReferences eMethods 2. Survey Questions [file jamahealthforum-e240139-s001.pdf]

Supplemental Online Content

Frank E, Zhao Z, Fang Y, et al. Trends in sexual harassment prevalence and recognition during intern year. *JAMA Health Forum*. 2024;5(3):e240139. doi:10.1001/jamahealthforum. 2024.0139

eMethods 1. Sample Weighting Strategy

eReferences

eMethods 2. Survey Questions

This supplemental material has been provided by the authors to give readers additional information about their work.

© 2024 Frank E et al. *JAMA Health Forum.*

eMethods 1. Sample Weighting Strategy

In accordance with previously described methods, we used a sample weighting strategy to reduce the potential bias introduced from differences between participants and nonparticipants and between participants who completed the sexual harassment questions and those who did not.[1]

To reduce potential bias owing to sampling, we used data from the Association of American Medical Colleges (AAMC) on the overall characteristics of first-year residents in the US in 2016, 2017, and 2021 as the reference target to correspond with our study cohorts. At the time of analysis, 2022 AAMC data was not yet available, therefore 2021 AAMC was utilized to correspond with the 2022 cohort. We performed a 2-step post-stratification ranking on the study data to construct weights such that the distribution of cohort year, sex, specialty, and race in the sample matched the AAMC data distribution. The first step was to generate between-cohort weights (w1b) with the raking variable to be cohort year; the second step was to generate weights within each cohort (w1w) with the raking variables to be sex, specialty and race. To account for differences between participants who completed the sexual harassment questions and those who did not, we calculated probability of participation, propensity score (PS), using the PSMATCH SAS procedure (SAS, version 9.4 [SAS Institute]) with variables correlated with the completion (cohort year, sex, specialty, race, baseline PHQ-9 depression score, early family environment score, and neuroticism score). The overlap weights (w2) for each person who completed the sexual harassment questions was $(1 - \mathrm{PS})$.[2]

The final weights were the product of post-stratification weights and overlap weights (w1b × w1w × w2).

© 2024 Frank E et al. *JAMA Health Forum.*

eReferences

1. Fang Y, Bohnert AS, Pereira-Lima K, et al. Trends in depressive symptoms and associated factors during residency, 2007 to 2019: A repeated annual cohort study. *Ann. Intern. Med.* 2002;175(1):56-64.
2. Fan L, Thomas LE, Fan L. Addressing extreme propensity scores via the overlap weights. *Am. J. Epidemiol.* 2019; 188(1):250-257.

© 2024 Frank E et al. *JAMA Health Forum.*

eMethods 2. Survey Questions.

WORK PLACE EXPERIENCE

We would like to ask you some questions related to sexual harassment. Some of these questions may be sensitive, but please be assured that your responses will be confidential.

Since starting your residency, were you ever sexually harassed?

- ❍ Yes
- ❍ No

If yes, how were you harassed? ____

Please read each of the situations listed and then check the box that matches how often you have had this experience. Some questions may appear repetitive, but please answer them despite this.

© 2024 Frank E et al. *JAMA Health Forum.*

Since starting your residency, have you ever been in a situation where any individuals…	Never	Once	Some times	Often	Very Often
1. Habitually told suggestive stories or offensive jokes?	❍	❍	❍	❍	❍
2. Made unwanted attempts to draw you into a discussion of personal or sexual matters (e.g., attempted to discuss or comment on your sex life)?	❍	❍	❍	❍	❍
3. Made crude and offensive sexual remarks, either publicly (e.g., in the office), or to you privately?	❍	❍	❍	❍	❍
4. Treated you “differently” because of your sex (e.g., mistreated, slighted, or ignored you)?	❍	❍	❍	❍	❍
5. Gave you unwanted sexual attention?	❍	❍	❍	❍	❍
6. Displayed, used, or distributed sexist or suggestive materials (e.g., pictures, stories, or pornography)?	❍	❍	❍	❍	❍
7. Frequently made sexist remarks (e.g., suggesting that women are too emotional to be scientists or that men should not be the primary caretakers of children because they are not nurturing)?	❍	❍	❍	❍	❍
8. Attempted to establish a romantic relationship with you despite your efforts to discourage this person?	❍	❍	❍	❍	❍
9. “Put you down” or was condescending to you because of your sex?	❍	❍	❍	❍	❍
10. Has continued to ask you for a date, drinks, dinner, etc., even though you have said “no”?	❍	❍	❍	❍	❍
11. Made you feel like you were being subtly bribed with some sort of reward or special treatment to engage in sexual behavior?	❍	❍	❍	❍	❍
12. Made you feel subtly threatened with some sort of retaliation for not being sexually cooperative (e.g., the mention of an upcoming evaluation, review, etc.)?	❍	❍	❍	❍	❍
13. Touched you (e.g., laid a hand on your bare arm, put an arm around your shoulders) in a way that made you feel uncomfortable?	❍	❍	❍	❍	❍
14. Made unwanted attempts to stroke or fondle you (e.g., stroking your leg or neck, etc.)?	❍	❍	❍	❍	❍
15. Made unwanted attempts to have sex with you that resulted in you pleading, crying, or physically struggling?	❍	❍	❍	❍	❍
16. Implied faster promotions or better treatment if you were sexually cooperative?	❍	❍	❍	❍	❍
17. Made it necessary for you to respond positively to sexual or social invitations in order to be well-treated on the job?	❍	❍	❍	❍	❍
18. Made you afraid you would be treated poorly if you didn’t cooperate sexually?	❍	❍	❍	❍	❍
19. Treated you badly for refusing to have sex?	❍	❍	❍	❍	❍

© 2024 Frank E et al. *JAMA Health Forum.*
