# Supplementary material for: Trends in Sexual Harassment Prevalence and Recognition During Intern Year
Source: JAMA Health Forum. 2024 Mar 22;5(3):e240139. doi: 10.1001/jamahealthforum.2024.0139 (PMC10960195; doi:10.1001/jamahealthforum.2024.0139)
Supplement: Supplement 2. — Data Sharing Statement [file jamahealthforum-e240139-s002.pdf]

Data Sharing Statement

Frank. Trends in Sexual Harassment Prevalence and Recognition During Intern Year. *JAMA Health Forum*. Published March 22, 2024. doi:10.1001/jamahealthforum.2024.0139

Data
Data available: Yes
Data types: Deidentified participant data
How to access data: Data are identified through ICPSR:
https://www.openicpsr.org/openicpsr/project/129225/version/V1/view
When available: With publication

Supporting Documents
Document types: None

Additional Information
Who can access the data: researchers whose proposed use of the data has been approved
Types of analyses: specified purpose
Mechanisms of data availability: Deidentified data will be publicly available via ICPSR
https://www.openicpsr.org/openicpsr/project/129225/version/V1/view
